# Occlusional Modifications Reversibly Alter Aquaporin 5 Expression and Localization in Rat Salivary Glands

**DOI:** 10.3389/fphys.2020.00528

**Published:** 2020-06-10

**Authors:** Eri Saito, Ippei Watari, Mariko Mizumachi-Kubono, Sumire Hsu-Hayashi, Takashi Ono

**Affiliations:** Department of Orthodontic Science, Graduate School of Medical and Dental Sciences, Tokyo Medical and Dental University, Tokyo, Japan

**Keywords:** aquaporin 5, immunohistochemical staining, occlusion, submandibular gland, incisor biteplate

## Abstract

**Background:**

Aquaporin 5 (AQP5) is a water channel–forming protein that plays a key role in saliva secretion. A decrease in masticatory function associated with the molar extraction adversely affects the submandibular salivary gland (SMG) in rats, inducing hypertrophic changes in the acinar cells and the expression of AQP5 in acinar cells or intercalated duct of the SMG. However, changes in AQP5 expression and localization in the SMG in association with occlusal modification have not been fully characterized.

**Methods:**

We examined the influence of the decline and recovery of masticatory function on expression and localization of AQP5 in the rat SMG by inserting and removing an incisor bite plate (IBP). Thirty 5-week-old male Wistar rats were randomly divided into IBP (*n* = 12), recovery (REC) (*n* = 6), and control (CON) (*n* = 12) groups. Each rat in both the IBP and REC groups was fitted with the IBP on its maxillary incisors. Rats without the IBPs served as controls. All rats were fed powder diet and water *ad libitum*. Rats in the IBP and CON groups were sacrificed after 14 (*n* = 6) and 28 (*n* = 6) days after the IBP attachment. In the REC group, the IBP was detached on the 14th day and sacrificed on 28th day after the IBP attachment. *AQP5* mRNA expression was quantified by reverse transcription–polymerase chain reaction. Changes in the localization of AQP5 were tracked by immunohistochemical staining.

**Results:**

Attachment of IBP resulted in a decrease in the expression of AQP5 in the IBP group. Changes in the localization of AQP5 were observed between 14 and 28 days in the IBP group. In contrast, changes in the expression and localization of AQP5 were not observed in the REC group.

**Conclusion:**

Findings suggested that a loss of molar occlusion, due to the IBP attachment, altered AQP5 expression and localization in the rat SMG. However, removal of the bite plate allowed the recovery of both AQP5 expression and its normal localization in the SMGs.

## Introduction

Saliva is important for the maintenance of the ambience and function of the oral cavity. Saliva has antibacterial, mucosal-protective, and bolus-forming activities. Thus, severe salivation disorders cause caries, oral infections, dysphagia, and aspiration pneumonia, resulting in a decrease in the quality of life (Hayashi et al., 2009; Vissink et al., 2010; Millsop et al., 2017). The function and morphology of the salivary glands are influenced by multiple factors, including systemic disease, drug side effects, radiation, and aging. Patients with Sjögren syndrome exhibit acinar atrophy and interstitial fibrosis (Daniels, 1984). Atrophy of the acinar cells, fat degeneration, and decreased saliva secretion have been observed in diabetic rats (Mahay et al., 2004). In rats, irradiation of the salivary glands causes atrophy of the acinar cells, dilation of the conduits, and a decrease in salivary flow (Marie et al., 2015). The number of acini decreases with age, with a concomitant increase in fatty and fibrous tissue (Liu et al., 2000).

Several reports have documented changes in the function and morphology of salivary glands in response to changes in the masticatory environment. When rats have been provided with an atypical hard bulk diet, an increase in the size of the parotid and submandibular glands, elevated amylase content of saliva, and changes in gene expression were observed. Conversely, there is a significant decrease in these indices when liquid or powder foods are provided during breeding (Johnson, 1982). Bulk diets stimulate increased saliva secretion, whereas liquid and powder diets decrease the volume of saliva secretion (Scott et al., 1990). Intriguingly, several reports have indicated that salivary gland morphology and function are restored when reintroducing solid foods. Salivary gland weight, neurotransmitter concentration, and saliva secretion recover nearly to the levels of rats fed with solid food following the change from liquid to solid diet. These indicate that the masticatory environment plays an important role in the maintenance of salivary gland function (Hall and Schneyer, 1964).

In rats, there are three major salivary glands: the parotid, submandibular, and sublingual glands. Additionally, a few small salivary glands are scattered on the oral mucosa and tongue. Protein and water secretions are performed in the salivary glands, with water constituting 99% of saliva. Therefore, secretion and transportation of water are considered to be essential for secretion of saliva. Salivation is controlled by the parasympathetic nervous system; acetylcholine acts on muscarinic receptors of the acinar cells, raising the intracellular calcium concentration and causing the secretion of water, mainly through a water channel called aquaporin (AQP).

Aquaporins, which are encoded by 13 genes in humans, are membrane proteins that function as a water channel. They are widely distributed throughout the body. Aquaporins 1, 5, and 8 are present in the cell membranes of the salivary glands (Baoxue et al., 2005). In particular, AQP5 is abundant in the salivary glands and has been reported to be involved in salivary function. Aquaporin 5 is located on the apical side of the acinar cells in the salivary gland, in the intercellular secretory capillary, and in the luminal cytoplasm of the interstitial conduit. A previous study has reported that expression of AQP5 decreases in the submaxillary glands in response to radiation (Li et al., 2006). Decreased AQP5 expression in the salivary glands has also been reported in Sjögren syndromic patients (Wang et al., 2009). Further, changes in the distribution of the acinar cells to the basal side (Steinfeld et al., 2001) and reduction in saliva volume have been observed in AQP5 knockout mice (Ma et al., 1999).

Our previous study suggested that maxillary molar extraction induced acinar cell hypertrophy and dispersed AQP5 expression in rat submandibular salivary gland (SMG) (Mizumachi-Kubono et al., 2012). However, little information is available regarding the relationship between impaired occlusion and salivary gland function. To date, it has been known that an occlusal hypofunction model, including molar extraction, causes functional deterioration of the masticatory muscles, periodontal tissue, and peripheral nerves in the oral cavity (Kaneko et al., 2001; Nakamura et al., 2013). Additional reports suggest that impaired oral function can be improved by restoring occlusal function. However, to our knowledge, similar studies on salivary gland hypofunction resulting from reduced occlusal function have not been reported. Furthermore, it is not known whether anatomical and functional changes in salivary glands are reversed following the recovery of occlusal function.

In this study, we aimed to evaluate the effect of decreased masticatory function and recovery of secretory function on salivary gland function in rodents. For this, we fashioned a masticatory function recovery model, by attaching a removable IBP over the rat maxillary incisors and a metal cap on the mandibular incisors, which prevent occlusal contact at the molars while the IBP was in position. Subsequently, we examined the expression and distribution of AQP5 in the salivary glands.

## Materials and Methods

### Experimental Animals

Five-week-old male Wistar rats (*n* = 30) were randomly assigned to IBP (*n* = 12), recovery (REC; *n* = 6), and control (CON; *n* = 12) groups. All rats were fed a powdered diet (CE-2; CLEA, Tokyo, Japan) throughout the experimental period. In both the IBP and REC groups, an IBP and metal cap constructed from band material (0.180 × 0.005 inches; Rocky Mountain Morita Corp., Tokyo, Japan) were attached to the maxillary and mandibular incisors, respectively, using a light-curing composite resin (Clearfil Liner Bond II; Kuraray, Okayama, Japan) at the age of 5 weeks. The IBP was T-shaped with the maxillary incisors in the center, ensuring that the anterior teeth of the lower jaw made an early contact with the bite plate. The metal cap prevented any mandibular anterior tooth abrasion. Therefore, while the bite plate is being worn, there could be no occlusal molar contact. At 14 days, both the IBP and metal cap were removed from the rats in the REC group. Rats in both the CON and IBP groups were euthanized at 14 and 28 days. Rats in the REC group were euthanized at 28 days. Both SMGs were isolated immediately after sacrifice.

### Wet Weight of SMG

The isolated submandibular glands were immediately weighed for wet rain on both the left and right sides. Then, the right side of the SMG was fixed with mild form, and the left side frozen in RNA STAT-60 for reverse transcription–polymerase chain reaction (RT-PCR).

### Preparation of the SMGs for Histological Analysis

The isolated SMGs were fixed immediately by overnight immersion in 4% paraformaldehyde (PBS; Mildform^®^; Wako Pure Chemical Industries, Osaka, Japan) at 4°C. After washing with PBS, the samples were then embedded in paraffin according to the standard protocol (Feng et al., 2014). Serial 5-μm-thick sections were cut, prepared for histological observation, and mounted on glass slides, as described below.

### Hematoxylin-Eosin Staining and Morphological Evaluation

For morphometric analyses, sections were deparaffinized with xylene and rehydrated in a graded ethanol series. The sections were then stained with H&E and observed under light microscopy (Microphoto-FXA; Nikon, Tokyo, Japan) at ×400 magnification. Digital images were captured using a digital camera (DXM1200; Nikon). The area occupied by one acinus was measured using imaging software (ImageJ 1.44; National Institutes of Health, Bethesda, MD, United States). The number of acinar cells in each acinus was determined by counting the number of acinar nuclei. The area occupied by one acinar cell was calculated by dividing the acinar area by the number of acinar cells in that acinus. To obtain mean values, 10 records per sample for each gland were analyzed in randomly selected microscopic fields and measured using digital imaging fields. All samples were analyzed in a blind fashion.

### Immunohistochemical Staining of AQP5 and Histological Evaluation

To evaluate AQP5 expression in the SMGs, we performed immunohistochemical staining using the three-step streptavidin–biotin–peroxidase method (VECTASTAIN ABC Staining Kit; Vector Laboratories, Burlingame, CA, United States). Briefly, sections were deparaffinized with xylene and rehydrated in a graded ethanol series. To activate the antigen, we used microwave treatment with unmasking solution (Antigen Unmasking Solution; Vector Laboratories). The solution was prewarmed for 10 min using three treatments of microwave irradiation 5 min each, followed by incubation at room temperature (RT) for 20 min. Endogenous peroxidase was blocked with peroxidase-blocking solution (Agilent Technologies, Santa Clara, CA, United States) for 10 min at RT. To prevent non-specific binding of antibodies, we incubated the sections with PBS containing 1% bovine serum albumin for 30 min at RT. Next, the sections were incubated for 1 h at RT with an anti-AQP5 primary antibody (#ab104751; Abcam, Cambridge, MA, United States) diluted 1:300 in PBS containing 0.3% Triton X-100 and 0.1% bovine serum albumin. The sections were then incubated with a diluted secondary anti-goat immunoglobulin G antibody (VECTASTAIN ABC Staining Kit; Vector Laboratories) for 30 min at RT.

After treatment with an avidin–biotin macromolecular complex (VECTASTAIN ABC Staining Kit; Vector Laboratories) for 30 min at room temperature and incubation with 3,3-diaminobenzidine for 1 min, the sections were washed in PBS, counterstained with hematoxylin, rinsed for 30 min under running tap water, and mounted with Mount-Quick “Aqueous” (Cosmo Bio Inc., Tokyo, Japan). We observed two sections per SMG sample and 20 fields of 200 × 200 pixels per section under ×400 magnification using light microscopy (DS-Ri1; Nikon). All procedures for image capture, identification, and processing were standardized before images were captured. The integral OD of each scanned image was computerized using a digital image analyzer in ImageJ 1.44 after the immunohistochemical stained area was extracted using Photoshop (Adobe Systems, San Jose, CA, United States), and the mean OD was obtained.

### Quantitative Analysis of AQP5 mRNA (RT-PCR)

Submandibular salivary gland tissues for RNA preparation were quickly separated and frozen at −80°C. Total RNA was isolated using RNA STAT-60^TM^ (Tel-Test Inc., Friendswood, TX, United States) reagent according to the instructions provided by the manufacturer. The cDNA was synthesized from 5.0 μg of total RNA using ReverTra Ace^TM^ qPCR RT Master Mix (TOYOBO, Osaka, Japan) under the conditions suggested by the manufacturer. Quantitative RT-PCR was conducted with a 7500 Real-Time PCR System (Applied Biosystems, Foster City, CA, United States) using KAPA SYBR^®^ FAST qPCR Kits (KAPA BIOSYSTEMS, Cape Town, South Africa). The following oligonucleotide primers for rat β-actin, AQP5, were purchased from Thermo Fisher Scientific (Waltham, MA, United States): β-actin 5′-TCTGGTCGTACCACTGCATT-3′ (forward) and 5′-AGACGCAGATGCATGAG-3′ (reverse), AQP5 5′-CCCTGCTGTCATGAA-3′ (forward) and 5′-CAGTCCTCCTCCGGCTCATA-3′ (reverse). All reactions for quantitative RT-PCR were carried out under the following parameters: 95°C for 3 min and 35 cycles of 95°C for 30 s, 60°C for 45 s, and 72°C for 15 s. Reverse transcriptase–PCR was performed three times for each primer, and the averaged results were used for statistical analysis. Relative mRNA expression was quantified using the 2^–ΔΔCT^ method with β-actin as an internal control. The mRNA expression levels are expressed as the n-fold difference relative to the mRNA expression in control rats.

### Statistical Analysis

All results are presented as means ± standard deviation. Data were analyzed after confirming whether the variance was equal in each group using the Barrett test; comparison between multiple groups was performed using the Tukey–Kramer test.

## Results

### Body Weight

**FIGURE 1:**
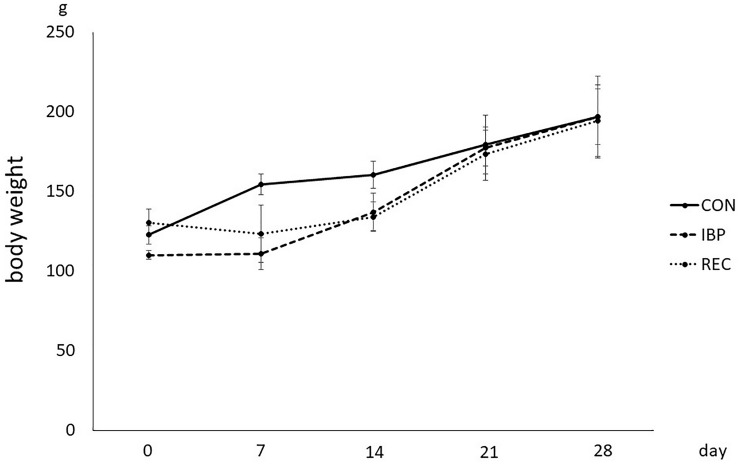
Body weight of the rat in CON, IBP, and REC group. Changes in body weights during the experimental period in CON, IBP, and REC groups. Data are presented as means ± standard deviation (*n* = 6 each).

The body weight of the rats in the IBP and REC groups began to decrease on the second day after attaching the IBP. On the fourth day in the IBP group, mean weight decreased to 8.65 g and decreased to 1.59 × 10^2^ g in the REC group. However, there were no significant differences in body weights between groups at 21 and 28 days (Figure 1).

### Wet Weight of SMG

**FIGURE 2:**
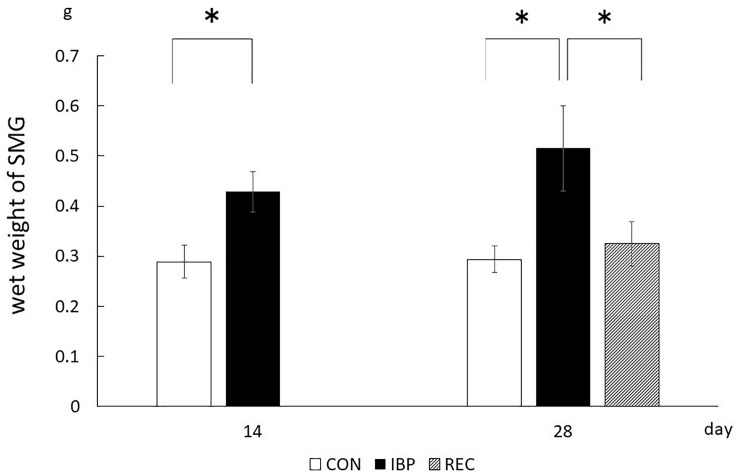
Wet weights of submandibular glands. The weight of the left and right submandibular glands of each rat was measured, and the average value was taken. The weight in the REC group was not significantly different from that in the CON group, whereas it was significantly increased in the IBP group. ^∗^*p* < 0.05.

The wet weight of SMGs in the CON group and IBP group was 2.89 × 10^–1^ ± 3.30 × 10^–2^ g and 4.28 × 10^–1^ ± 3.96 × 10^–2^ g, on day 14, respectively. On the 14th day after attachment of the IBP, the submandibular gland wet weight in the IBP group increased significantly compared to the CON group. The wet weight of salivary glands of CON, IBP, and REC group was 2.94 × 10^–1^ ± 2.65^–2^ g, 5.15 × 10^–1^ ± 8.49 × 10^–2^ g, and 3.25 × 10^–1^ ± 4.37 × 10^–2^ g, on day 28, respectively. On the 28th day, the IBP group’s submandibular gland wet weight further increased compared to the CON group. Regarding the REC group, the wet weight was the same as that of the CON group on the 28th day (Figure 2).

### Histological Findings of HE Staining

**FIGURE 3:**
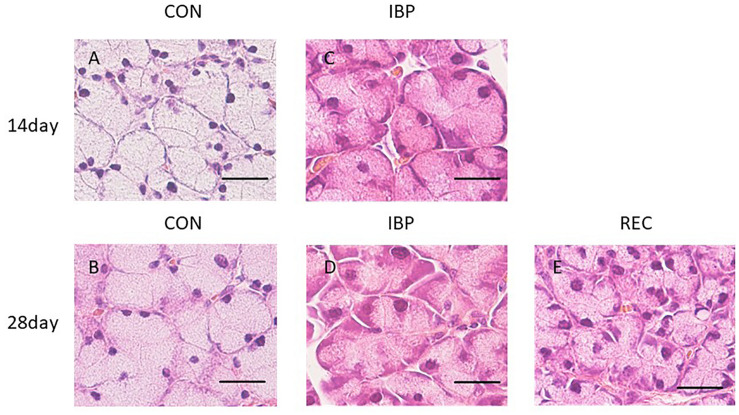
Hematoxylin-eosin staining of SMG at ×400 magnification with an optical microscope. Morphological observation of HE staining of the SMG in the CON group **(A,B)**, IBP group **(C,D)**, and REC group **(E)**. The IBP group at 14 days **(C)** showed an enlargement of the acini. At 28 days, the size of the acini decreased **(D)** compared to the size at 14 days. **(E)** In the REC group at 14 days, the size of the acini was approximately the same as that of the CON group **(E)**. H&E, hematoxylin and eosin; CON, control group; IBP, incisor bite plate group; REC, recovery group. Bar = 25 μm, ×400 magnification.

When observed at ×400 magnification with an optical microscope, acinar cells and ducts (intercalated, granulated, striated, and excretory) did not change with age in the CON group (Figure 3). In the IBP group, we observed marked hypertrophy of the acinar cells on 14th day after IBP attachment. This was accompanied by nuclear enlargement and thickening of myoepithelial cells. On the 28th day, the acinar cells shrank, but the nuclear enlargement and thickening of the myoepithelial cells persisted. In the REC group, a slight nuclear enlargement and thickening of myoepithelial cells were observed. However, the tissue exhibited normal properties when compared with the IBP group.

**FIGURE 4:**
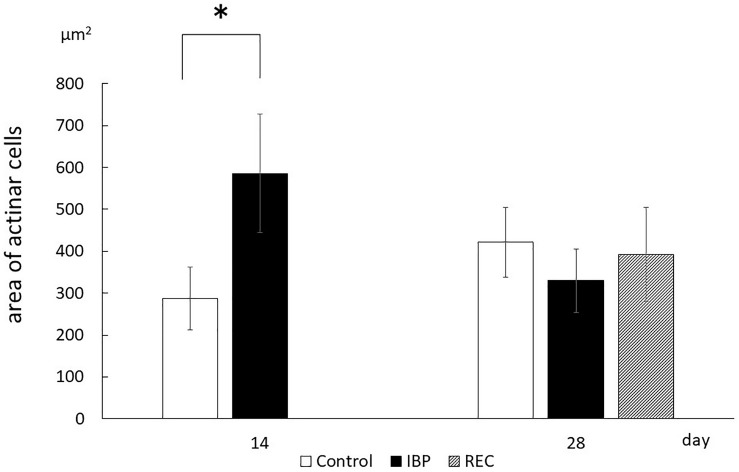
The area of acinar cell. Morphometric measurements of acinar cells of the SMG. The area occupied by one acinar cell was calculated by dividing the acinar area by the number of acinar cells in that acinus. Data are presented as the mean ± standard deviation (*n* = 6 each, *p < 0.05).

Morphometric analysis showed that the cross-sectional area of acinar cells in the IBP group significantly (*p* < 0.05) increased compared with that of the CON group at 14 days. The acinar cell cross-sectional area significantly increased in the CON group between 14 and 28 days. Conversely, a significant decrease was observed in the IBP group over the same period. At 28 days, no significant differences in the cross-sectional area of acinar cells were observed among the control, IBP, and REC groups (Figure 4).

### Expression and Distribution of AQP5 in SMG

**FIGURE 5:**
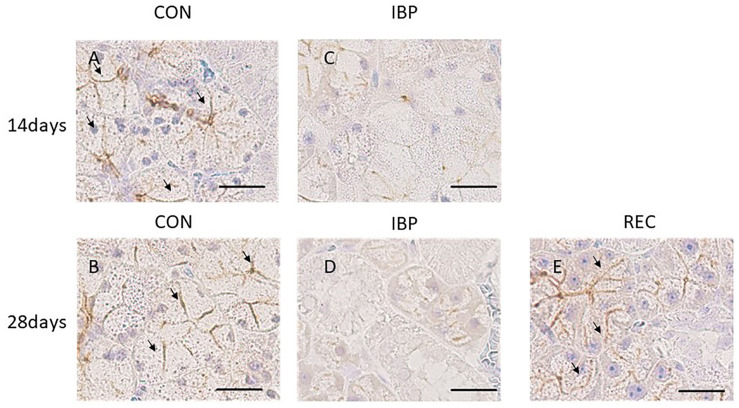
Immunohistochemical images of AQP5 expression. **(A,B)** CON group; AQP5 was detected on the apical plasma membrane (APM) and in the intercellular secretory canaliculi of acinar cells and on the luminal side of the intercalated duct. **(C,D)** IBP group; immunostaining of AQP5 in the APM and intercellular secretory canaliculi of acinar cells; almost no staining was observed in the APM of the acinar cells. **(E)** REC group; AQP5 was detected at the same level as that of the CON group. AQP5, aquaporin 5; CON, control group; IBP, incisor bite plate group; REC, the recovery group.

The specificity of AQP5 immunohistochemical staining was demonstrated using the non-immune control, which did not exhibit non-specific staining. In normal salivary glands, AQP5 was localized to the APM of the acinar cells, intercellular canaliculi, and the luminal cytoplasm of the intercalated ducts. In the CON group, AQP5 was localized to the APM, intercellular canaliculi, and luminal side of the intercalated duct during all experimental periods, but not to the BPM, cytoplasm or nucleus of acinar cells, the granular conduit, the striated duct, or the excretory duct (Figure 5). In the IBP group, we observed faint staining and the changes of expression and localization in AQP5. The localization of AQP5 to the APM and intercellular canaliculi was rarely observed on days 14 and 28. On the other hand, in the REC group, AQP5 localization was confirmed in the APM of the acinar cell, intercellular canaliculi, and luminal side of the intercalated duct. Further, expression recovered to the same level as that of the CON group.

**FIGURE 6:**
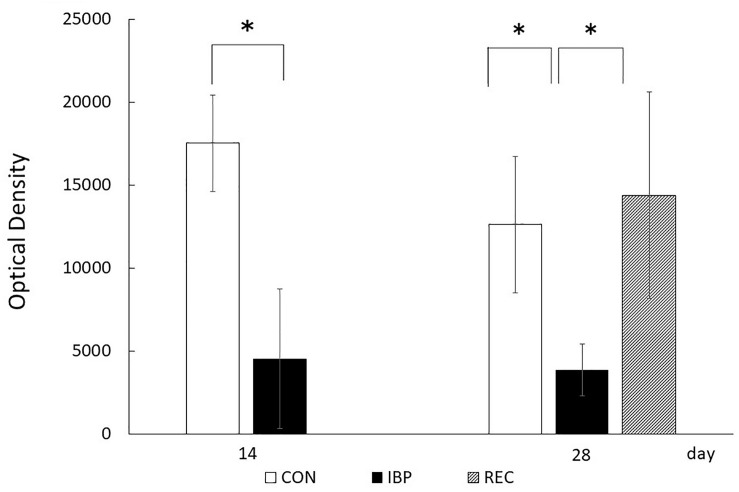
Optical density (OD) of AQP5 immunohistochemical positive staining. The procedures for anti-AQP5 immunohistochemical image capturing, identification, and processing were standardized before images were captured. Concerning positive staining of AQP5, the integral OD of each scanned image was computerized using a digital image analyzer in ImageJ 1.44 after the immunohistochemical stained area was extracted using Photoshop (Adobe Systems), and the mean OD was obtained. Data are presented as means ± standard deviation (*n* = 6, each group; *p < 0.05).

The respective OD values for the CON, IBP, and REC groups were 1.75 × 10^4^ ± 2.89 × 10^3^ and 4.33 × 10^3^ ± 4.21 × 10^3^ on the 14th day. On the 28th day, they were 1.26 × 10^4^ ± 4.13 × 10^3^, 3.86 × 10^3^ ± 1.58 × 10^2^, and 1.43 × 10^4^ ± 6.21 × 10^3^. A marked decrease in OD in the IBP group was observed on days 14 and 28 compared with the CON group. In contrast, there was no significant difference in OD values between the REC group and the CON group (Figure 6).

### Quantitative Analysis of AQP5 mRNA (RT-PCR)

**FIGURE 7:**
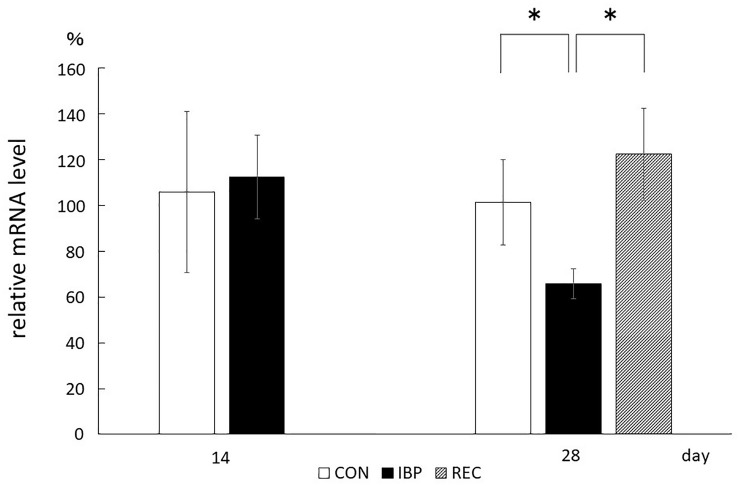
Quantitative analysis of AQP5 mRNA (qPCR). Aquaporin 5 mRNA prepared from submandibular gland tissues was analyzed by RT-PCR using primers specific for AQP5. Aquaporin 5 mRNA levels normalized to β-actin. Data are means ± SE. ^∗^*p* < 0.05.

From the RT-PCR data, relative mRNA levels for AQP5 were 1.06 ± 3.52 × 10^–1^ in the CON group and 1.13 ± 1.83 × 10^–1^ in the IBP group. There were no significant differences observed between groups in the amount of AQP5 mRNA on the 14th day. On day 28, the levels were 1.01 ± 1.87 × 10^–1^ in the CON group, 6.57 × 10^–1^ ± 6.58 × 10^–2^ in the IBP group, and 1.22 ± 2.02 × 10^–1^ in the REC group. There was significantly less AQP5 expression in the IBP group. On day 28, the AQP5 mRNA level in the IBP group was significantly decreased relative to the other two groups; however, there was no significant difference between the mRNA levels of the CON and REC groups (Figure 7).

## Discussion

Mastication is the critical importance of maintaining physiological function in stomatognathic system. The animal models of occlusal hypofunction, tooth extraction (Ejiri et al., 2006), feeding the soft/liquid diet (Mavropoulos et al., 2008), or resection of the masseter (Yonemitsu et al., 2007) are common; however, to avoid surgical stress and to allow recovery from occlusal hypofunction, we used IBP for making the occlusal modification in this study. We attached the IBP to the maxillary and mandibular incisors for creating occlusal hypofunction, preventing molar contacts during the entire experimental period. We set and detached the IBP non-invasively for allowing recovery. The body weights of the rats in the CON, IBP, and REC groups were measured during the experimental period. No significant differences were observed among the rats in the three groups at 0, 7, 14, 21, and 28 days with IBP attachment, so there was no apparent alteration of general metabolism.

After 14 days of IBP attachment, the wet weights of SMGs of the IBP group were significantly higher than those of the CON group. Histological observation showed that the hypertrophy of acinar cell area that increased the width of myoepithelial cells decreased significantly and enlargement of the nucleus of acinar cell observed in IBP group. Immunohistochemistry revealed that functional localization of AQP5 in acinar cells was not observed in the IBP group; however, the amount of AQP5 mRNA was different between the CON and IBP groups. These changes have also been observed in other hypofunctional animal models involving maxillary molar extraction (Mizumachi-Kubono et al., 2012) or repeated amputation of incisors (Wells et al., 1959; Takeda et al., 1986). The alterations in our experiment were smaller than those in these previous hypofunctional models were, but showed the same pattern. The reduction of afferent input from periodontal ligament of molars (Kaneko et al., 2001), alteration of autonomic activity affected by occlusal stimuli (Proctor and Carpenter, 2007), and the change of the jaw movement (Gavião et al., 2004) may influence SMG function in the IBP group. The SMG degeneration in this study is thought to be caused by reduction of water secreting function via AQP5 in acini due to impaired masticatory function.

On the other hand, at 28 days after IBP attachment, further histological degeneration of acinar cells, wet weight of SMG, and qualitative and quantitative reduction of AQP5 expression were observed in the IBP group relative to the CON group. Furthermore, normalization of functional localization of AQP5 and quantitative recovery of AQP5 mRNA were confirmed. These results showed that SMG degeneration induced by IBP attachment can be restored, especially the negative impact on AQP5, which plays a critical role in water transport in acini. This is the first report to indicate that salivary gland dysfunction associated with occlusal alteration can be restored. One possible reason for our observed restoration is the age of the rats. Functional maturation of SMG is known to continue to 10 weeks after birth (Jacoby and Leeson, 1959). In this experiment, we used younger rats with less-mature SMGs. Further studies are needed to examine the effects of aging.

In addition, the removal of IBP had improved the dryness of the mouth, which might have contributed to the histological recovery of SMG observed in the REC group. With IBPs on the incisors, it was difficult for the rats to close their mouths. The improvement of mouth dryness after IBP removal led to normalization of jaw movement, which might have helped in the recovery of SMG in REC group.

One limitation of our study is the uncertainty regarding the extent to which these results correspond to humans. Although there are some anatomical similarities between human and rat, differences do exist, such as the highly developed interstitial ducts in rat SMG. Another limitation is that, in our study, the functions other than water transportation in SMG were not investigated.

Our findings provide support for the hypothesis that the occlusal modification has an effect on salivary gland function. Clinical studies have shown that occlusal hypofunction causes a series of salivary disorders (Matsuda et al., 2009). This study supports these findings in terms of water transportation in salivary gland. The findings of our study also emphasize the importance of preventing malocclusion for maintaining physiologically normal saliva function.

## Conclusion

The structure and water secretion function of the submandibular glands were significantly altered due to IBP-induced decrease in masticatory function. Removal of the bite plate allowed the recovery of both AQP5 expression and its normal localization in the SMGs, thus restoring the masticatory function.

## Data Availability Statement

The datasets generated during the current study are available from the corresponding author on reasonable request.

## Ethics Statement

All experimental procedures were approved by the Institutional Animal Care and Use Committee (#0170353C2) and performed in accordance with the Animal Care Standards of Tokyo Medical and Dental University.

## Author Contributions

ES contributed to the design of the study, animal handling, data acquisition, data interpretation performed the statistical analysis, and drafted the manuscript. IW contributed to the design of the study, data interpretation, and participated in manuscript formatting. MM-K conceived the study. SH-H participated in the design of the study and data interpretation. TO participated in manuscript design and formatting. All authors read and approved the final manuscript.

## Conflict of Interest

The authors declare that the research was conducted in the absence of any commercial or financial relationships that could be construed as a potential conflict of interest.

## Abbreviations

APM: apical membrane
AQP5: aquaporin 5
BPM: basolateral membrane
H&E: hematoxylin and eosin
IBP: incisor bite plate
OD: optical density
PBS: phosphate-buffered saline.
